# CHIP phosphorylation by protein kinase G enhances protein quality control and attenuates cardiac ischemic injury

**DOI:** 10.1038/s41467-020-18980-x

**Published:** 2020-10-20

**Authors:** Mark J. Ranek, Christian Oeing, Rebekah Sanchez-Hodge, Kristen M. Kokkonen-Simon, Danielle Dillard, M. Imran Aslam, Peter P. Rainer, Sumita Mishra, Brittany Dunkerly-Eyring, Ronald J. Holewinski, Cornelia Virus, Huaqun Zhang, Matthew M. Mannion, Vineet Agrawal, Virginia Hahn, Dong I. Lee, Masayuki Sasaki, Jennifer E. Van Eyk, Monte S. Willis, Richard C. Page, Jonathan C. Schisler, David A. Kass

**Affiliations:** 1 Division of Cardiology, Department of Medicine, Johns Hopkins Medical Institutions, Baltimore, MD 21205 USA; 2 Cedar Sinai Medical Center, Advanced Clinical Biosystems Research Institute, The Smidt Heart Institute, 8700 Beverly Blvd, AHSP A9229, Los Angeles, CA 90048 USA; 3 Division of Cardiology, McAllister Heart Institute, The University of North Carolina at Chapel Hill, Chapel Hill, NC 27599 USA; 4 Department of Pharmacology, The University of North Carolina at Chapel Hill, Chapel Hill, NC 27599 USA; 5 Department of Chemistry and Biochemistry, Miami University, Oxford, OH 45056 USA; 6 Present Address: Division of Cardiology, Department of Medicine, Medical University of Graz, 8036 Graz, Austria

**Keywords:** Mechanisms of disease, Phosphorylation, Myocardial infarction

## Abstract

Proteotoxicity from insufficient clearance of misfolded/damaged proteins underlies many diseases. Carboxyl terminus of Hsc70-interacting protein (CHIP) is an important regulator of proteostasis in many cells, having E3-ligase and chaperone functions and often directing damaged proteins towards proteasome recycling. While enhancing CHIP functionality has broad therapeutic potential, prior efforts have all relied on genetic upregulation. Here we report that CHIP-mediated protein turnover is markedly post-translationally enhanced by direct protein kinase G (PKG) phosphorylation at S20 (mouse, S19 human). This increases CHIP binding affinity to Hsc70, CHIP protein half-life, and consequent clearance of stress-induced ubiquitinated-insoluble proteins. PKG-mediated CHIP-pS20 or expressing CHIP-S20E (phosphomimetic) reduces ischemic proteo- and cytotoxicity, whereas a phospho-silenced CHIP-S20A amplifies both. In vivo, depressing PKG activity lowers CHIP-S20 phosphorylation and protein, exacerbating proteotoxicity and heart dysfunction after ischemic injury. CHIP-S20E knock-in mice better clear ubiquitinated proteins and are cardio-protected. PKG activation provides post-translational enhancement of protein quality control via CHIP.

## Abstract (web summary)

Carboxyl terminus of Hsc70-interacting protein (CHIP) is proteostasis regulator. Here the authors show that CHIP-mediated protein turnover is enhanced by PKG-mediated phosphorylation, which results in attenuated cardiac ischemic proteotoxicity.

## Introduction

The ubiquitin proteasome system (UPS) regulates post-transcriptional protein stability by identifying and then either repairing or disposing of damaged/misfolded proteins^1^. The degradation process employs a three-enzyme cascade (the E1 ubiquitin-activating enzymes, E2 ubiquitin-conjugating enzymes, and E3 ubiquitin ligases) that ultimately conjugate multiple molecules of ubiquitin, an 8 kDa protein, to selective lysine residues on a target protein. The poly-ubiquitin chain is recognized by the 26S proteasome, and the ubiquitinated substrate is then degraded and recycled back into amino acids^1^. In addition to having a constitutive role in protein homeostasis, the UPS is actively engaged in stress conditions where misfolded/damaged proteins accumulate, aggregate, and can cause/exacerbate disease^2^. Such proteotoxicity contributes to a variety of heart diseases, including myocardial ischemia/reperfusion/infarction^3^ and amyloidosis, and neurological disorders such as Alzheimer’s and Parkinson’s disease^4^.

The carboxyl terminus of Hsc70-interacting protein or CHIP (encoded by the gene known as *Stub1*) is an important member of the UPS system, functioning as both an E3-ligase and co-chaperone, and facilitating protein degradation^5^. CHIP is ubiquitously expressed, with the highest protein levels found in hypermetabolic tissues such as cardiac and skeletal muscle. The linear structure of CHIP is comprised of triple tandem tetratricopeptide repeat (TPR)-domains in the N-terminal, a central coiled-coil domain, and a C-terminal U-box domain that confers its ligase activity^6^. The TPR domain and the adjacent charged region of ~200 amino acids form the binding site to heat shock proteins (HSP), principally Hsc70, Hsp70, and Hsp90. HSP-CHIP interactions can modify substrate targeting and can suppress chaperone-refolding activity, ultimately transforming the machinery to one favoring protein destruction and recycling^7,8^. CHIP mediates protein degradation via the proteasome, as well as by autophagy-lysosome-dependent pathways^9^. Studies using CHIP genetic loss of function models report worsened heart responses to hemodynamic or ischemic stress^10,11^, accelerated aging coupled to insulin receptor dysregulation^12^, and exacerbated neurodegenerative disease^13,14^ and oncogenesis^15^. CHIP gene upregulation has been protective against such disorders^16–18^.

In contrast to artificial gene upregulation, post-translational control of CHIP functionality remains poorly understood. One effector is mono-ubiquitination that enhances CHIP substrate targeting for degradation^19^. CHIP phosphorylation is also detected in proteomics databases^20,21^, but whether this has a broad modulatory role is unknown. Protein kinase G (PKG) is the primary effector for the second messenger cyclic guanosine monophosphate (cGMP) generated by nitric oxide or natriuretic peptide stimulation. In proteotoxicity models of heart muscle^22,23^ and neurodegenerative disease^24^, PKG activation stimulates 26S proteasomes and reduces misfolded protein aggregation and associated proteotoxicity. This happens rapidly and impacts both short-lived and long-lived proteins^24^. We hypothesized that PKG may impact more than the proteasome by also modifying chaperone/E3-ligase functionality related to CHIP.

Here, we show that CHIP is phosphorylated by PKG at a highly conserved serine 20 (mouse; S19 human), enhancing CHIP functionality principally by increasing its post-translational half-life and protein interaction with Hsc70. Inhibiting PKG activation in myocytes and intact hearts that were then subjected to ischemic stress results in lower CHIP protein levels and its S20 phosphorylation, coupled to ubiquitinated protein accumulation with worse cytotoxicity or organ dysfunction. The opposite occurs if PKG is activated or CHIP mutated to a phosphomimetic (S20E). In vivo, mice with a CHIP S20E KI mutation are protected against myocardial infarction with improved protein quality control (PQC), smaller infarcts and enhanced function. These data reveal substantial potency to CHIP S20 (S19, human) phosphorylation as a means of protecting against damaged /misfolded protein accumulation and associated toxicity.

## Results

### PKG activity regulates PQC in a CHIP dependent manner

**Fig. 1:**
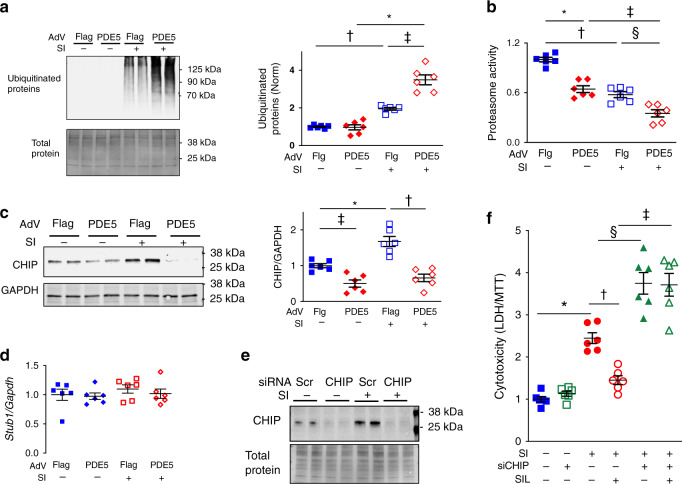
Effect of simulated ischemia (SI)+/− PKG blockade on ubiquitinated proteins and CHIP. **a** Left: example immunoblot for ubiquitinated proteins from myocytes infected with adenovirus (AdV) for Flag or PDE5A-Flag (PDE5), the latter to suppress PKG activation (c.f. Supplementary Fig. 1a, b). Cells are then subjected to normal conditions or SI. Right**:** summary results, *n* = 6 biological replicates/group, 2WANOVA, Tukey multiple comparisons test (mct): *p < 10^−8^, ^†^*p* = 0.0014, ^‡^*p* = 4 × 10^−6^. **b** Blocking PKG activation by PDE5A*oe* reduces proteasome activity similarly at baseline or after SI (*n* = 6 biological replicates/group, 2WANOVA, Tukey mct: *p = 8 × 10^−6^, ^†^8 × 10^−7^, ^‡^1 × 10^−4^, ^§^0.002). **c** CHIP protein expression increases after SI but this is blocked by PDE5Aoe, 6 biological replicates/group, 2WANOVA, Tukey mct, *0.001, ^†^7 × 10^−6^, ^‡^0.015. **d** CHIP mRNA expression is unaltered between groups, six biological replicates/group; 1WANOVA, *p* = 0.7. **e** Immunoblot showing efficacy of CHIP protein suppression in basal and SI conditions after incubation with specific but not scrambled (Scr) silencing RNA (siRNA), summary in Supplementary Fig. 1c. **f** LDH/MTT ratio in NRCMs exposed to control or SI conditions, +/−siRNA to CHIP (100 pM) or Scr, and with or without PDE5A inhibitor, sildenafil (SIL, 1 µM). *n* = 6 biological replicates/group; 1WANOVA, Sidak mct: *p = 9 × 10^−6^, ^†^0.002, ^‡^1.2 × 10^−9^, ^§^5 × 10^−5^. Source data are provided as a Source Data file. Individual data and mean ± SEM shown in each summary panel.

To test the intrinsic role of PKG activation on PQC, we first performed loss of function studies in cultured neonatal rat cardiomyocytes (NRCMs) transduced with an adenovirus expressing either epitope (Flag) tagged cGMP-selective phosphodiesterase 5 (PDE5A*oe*) to degrade cGMP and block PKG activation, or Flag alone (Supplementary Fig. 1a, b). Exposure to 48 h of simulated ischemia (SI; deoxyglucose + acidosis + hypoxia) increased ubiquitinated proteins (UP) that further rose in cells with PDE5A*oe* (Fig. 1a). PDE5*oe* also reduced proteasome activity in control myocytes and in those exposed to SI (Fig. 1b), the latter paralleling increased UP. Given the important role of CHIP in myocardial protein turnover, we measured steady-state levels of CHIP under these same conditions and found that while SI increased CHIP protein quantity, this was blocked by PDE5Aoe. PDE5*oe* reduced CHIP protein levels in controls as well, but this was amplified after SI (interaction *p* < 0.05, Fig. 1c). This change was post-translationally driven, as CHIP gene expression (*Stub1*) was unchanged in each condition (Fig. 1d).

To test the importance of PKG-CHIP modulation on PQC after SI, myocytes were transfected with CHIP-siRNA or a scrambled control-siRNA construct, and then co-treated with (or without) the PDE5A inhibitor sildenafil (SIL, 1 μM) which stimulates PKG by suppressing cGMP hydrolysis (Fig. 1e, Supplementary Fig. 2). SIL reduced SI-induced cytotoxicity in cells expressing CHIP, however this protective effect was lost in cells lacking CHIP (Fig. 1f). Moreover, silencing CHIP worsened cytotoxicity to SI. Thus, PKG assists intrinsic protein degradation by enhancing proteasome activity and by augmenting CHIP protein expression, reducing cytotoxicity with SI.

### PKG directly phosphorylates CHIP at Ser20 in cells

**Fig. 2:**
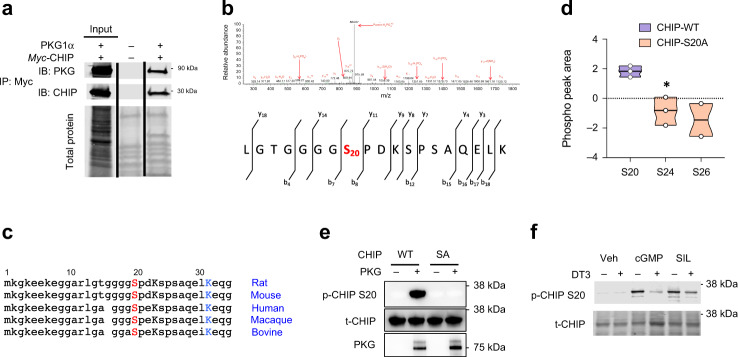
CHIP is phosphorylated by PKG at Serine 20. **a** Immunoprecipitation of Myc-tagged CHIP with PKG1α. Lanes: 1-input, 2 negative control, 3-IP signal after three bead washes. Experiment replicated ×3. **b** Mass spectroscopy detects increased Chip-S20 (mouse sequence) phosphorylation with acute PKG activation. The lower schematic shows the sequence and fragmentation matching the spectra (above). The M2S spectra has red annotations for the assigned ion fragments, with three fragment ions localizing phosphorylation at S20 (b8 and y11 indicate it occurs on the N-terminal sequence of LGTGGGGS, b7 indicates the sequence LGTGGGG does not contain the site. Study performed with 3 biological replicates. **c** CHIP peptide sequence from start of N-terminus shows high level of conservation including three serines (S19, S23, S25 for human; S20, S24, S26 for mouse and rat). Lysine 30 (K30 human, K31 mouse) is also nearby, highly conserved, and known to be critical for CHIP-chaperone binding. **d** Mass Spec detection of serine phosphorylation of recombinant human CHIP peptide fragment containing residues 13–30 (Uniprot Q9UNE7). Only S20 phosphorylation is detected with WT CHIP. If S20A mutation is expressed, some reduced phosphorylation is observed at S24 and S26. All biological replicates shown in figure, truncated violin plot, median and 25/75%; unpaired *t*-test, *p = 0.01 vs CHIP-WT. **e** Recombinant PKG1α/CHIP assay shows pS20 signal by immunoblot for full length WT but not S20A mutant. Example of three biological replicates. **f** Phospho S20 antibody detects CHIP phosphorylation at S20 in rat myocytes exposed to cGMP or PDE5A inhibition, and both are blocked by concomitant PKG inhibition with DT3 (1 μM). Gel repeated ×3, with 3–4 biological replicates. Summary analysis in Supplementary Fig. 2a.

To determine if the involvement of PKG and CHIP occurs via direct protein-protein interactions, we expressed *myc*-tagged CHIP as bait in HEK293 cells and found PKG to be a co-immunoprecipitant (Fig. 2a). In a prior Mass Spec-derived database of proteins in adult rat myocytes subjected to 15 minutes PKG activation^25^, we found CHIP modified at S20 (S20 in rat and mouse, S19 human, Fig. 2b), a highly conserved residue in the TPR domain (Fig. 2c). Human CHIP peptide fragments containing amino acid residues 13–30 were incubated with cGMP/PKG and assayed by Mass Spec. We observed phosphorylation at S20 with wildtype (WT) CHIP but not a phospho-silenced CHIP-S20A mutation. Phosphorylation was also observed at neighboring S24 and S26, but only if a S20A CHIP mutant was used (Fig. 2d). This indicates the primary PKG-targeted serine on CHIP is S20. A monoclonal phospho-antibody generated against the S20 site in CHIP was tested in vitro with recombinant PKG1α and CHIP, verifying a consistent phospho-S20-CHIP signal. Use of a CHIP-S20A mutant CHIP confirmed specificity (Fig. 2e). CHIP pS20 increased in cardiomyocytes in which PKG was stimulated by addition of cGMP or PDE5 inhibition with SIL. Importantly, this was prevented by co-blockade of PKG activation by the peptide DT3 (1 μM, Fig. 2f, Supplementary Fig. 3). Thus, PKG interacts with and directly phosphorylates CHIP at serine 20.

### Genetic alteration of CHIP S20 alters ischemic proteotoxicity in myocytes in vitro

**Fig. 3:**
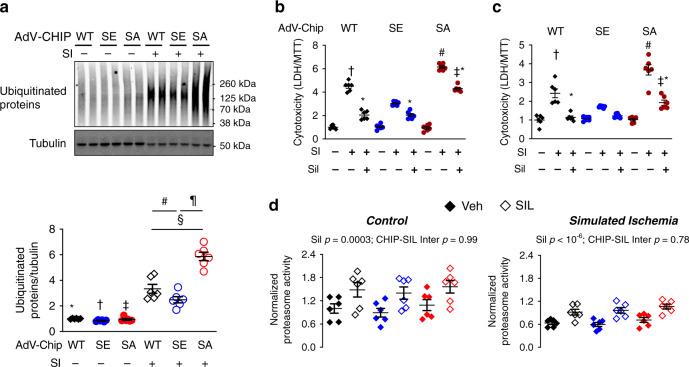
Functional regulation of UPS by Chip S20 is revealed by gain and loss of function phospho-mutants. **a** Upper: representative Western blot of ubiquitinated proteins in NRCMs infected with adenovirus expressing WT, S20E (SE), or S20A (SA) CHIP, and exposed to normal or SI conditions for 48 h; Lower: summary results, data normalized for protein loading, and to control WT-CHIP; three gels, six biological replicates, 2WANOVA, Tukey mct: *p = 7 × 10^−8^, ^†^3 × 10^−5^, ^‡^10^−9^, ^§^4 × 10^−8^, ^¶^<10^−9^, ^#^0.03. **b** Cytotoxicity (LDH/MTT assays) from SI is reduced by (SE) and increased by SA CHIP expression versus WT. PDE5A inhibitor sildenafil (SIL) reverses cytotoxicity in WT, but less so with SE or SA expression. 2WANOVA, 1.3 × 10^−5^ genotype condition interaction; Sidak mct: ^†^*p* = 1.3 × 10^−8^ vs SE-SI, 1.2 × 10^−9^ vs SA-SI; ^#^10^−11^ SE-SI; ^‡^<10^−11^ vs WT-SI + SIL and SE-SI+SIL; *p < 2 × 10^−5^ vs SI for each respective CHIP group. **c** Same experiment using AdV expressing combined (S20E, S24E; SE) or (S20A, S24A; SA). 2WANOVA, Sidak mct: ^†^*p* = 0.01vs SE-SI, 2 × 10^−5^ vs SA-SI; ^#^4.8 × 10^−9^ SE-SI; ^‡^0.03 vs SE-SI+SIL; *p* = 0.005 vs WT-SI + SIL; *1.4 × 10^−5^ vs WT-SI, 5.6 × 10^−8^, vs SA-SI. **d** Proteasome activity in resting NRCMs is augmented by PKG activation by PDE5 inhibitor sildenafil (SIL) in control myocytes and cells subjected to SI. Results of 2W-ANOVA for SIL effect and for interaction between SIL effect and gene-type of CHIP expressed are provided in the figures. *N* = 6 biological replicates for all conditions/groups. Source data are provided as a Source Data file. Individual data and mean ± SEM shown in each summary panel.

To directly probe the impact of S20 phosphorylation on proteotoxicity, we generated adenovirus expression vectors for CHIP-WT, or CHIP-S20E or CHIP-S20A. Myocytes were infected to express one of these forms, and then subjected to SI for 48 h. While each showed an increase in ubiquitinated proteins over non-stress controls, this was significantly amplified by CHIP-S20A and attenuated by CHIP-S20E expression compared to CHIP-WT (Fig. 3a). These differences were paralleled by disparities in SI-induced cytotoxicity (LDH/MTT ratio, Fig. 3b). Cells expressing CHIP-S20E mimicked the cyto-protection observed in CHIP-WT expressing myocytes co-incubated with the SIL to stimulate PKG. By contrast, cells expressing CHIP-S20A displayed greater cytotoxicity and blunted protective effects from SIL. Mutating only the neighboring serine (S24 mouse, S23 human) had negligible impact on cytotoxicity (Supplementary Fig. 4) and combining both S20 and S24 mutations yielded essentially the same response as with S20 modification alone (Fig. 3c). Thus, S20 is the primary effector of the cellular phenotype. The finding that SIL still increased proteasome activity regardless of whether WT or phospho-mutant CHIP was expressed for both control and SI conditions is consistent with PKG-stimulation of proteasome activity independent of CHIP^22^ (Fig. 3d).

### PKG activity increases CHIP phosphorylation and protein expression and counters proteotoxicity after myocardial infarction in vivo

**Fig. 4:**
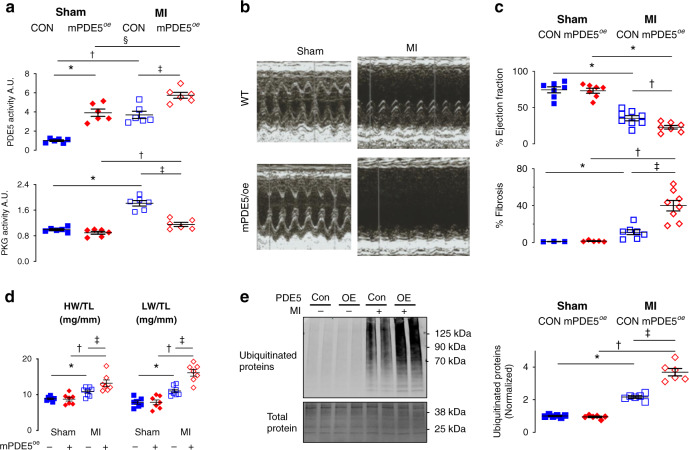
Suppressed myocyte PKG activity in vivo worsens infarct remodeling. **a** PDE5A and PKG enzyme activity in peri-infarct region at baseline and after myocardial infarction in mice with myocyte-specific PDE5A overexpression (mPDE5^oe^) versus littermate controls (CON). 2WANOVA, Tukey Post mct: Upper: *10^−5^, ^†^3 × 10^−5^, ^‡^8 × 10^−4^, ^§^2.6 × 10^−3^; Lower: *2 × 10^−8^, ^†^0.025, ^‡^9 × 10^−7^. **b** Example of M-mode echocardiograms in mPDE5/*oe* versus littermate controls 1-week following myocardial infarction (MI). **c** Summary data for left ventricular ejection fraction (EF, *n* = 7–8 mice/group) and for fibrosis (*n* = 3–8 biological replicates/group) for same study. For EF: Repeated measures mixed effects model, Holm-Sidak mct: *p < 2 × 10^−8^; ^†^*p* = 0.03; for fibrosis: biological replicates: 3–5 – sham, 7–8 MI; Brown-Forsythe Welch ANOVA, Dunnetts mct: *p = 0.02, ^†^0.008, ^‡^0.003. **d** Heart and Lung weight normalized to tibia length for same study (HW/TL, LW/TL respectively). *N* = 7–8 biological replicates; 2WANOVA, Sidaks mct: for HW/TL: *p = 0.04, ^†^2 × 10^−5^, ^‡^0.01; LW/TL: *0.003, ^†^8 × 10^−9^, ^‡^2 × 10^−5^. **e** Ubiquitinated protein increase after MI in the peri-infarct zone is greater in mPDE5/*oe* hearts. *N* = 6 biological replicates, 2WANOVA, Tukey mct: *8.6 × 10^−6^, ^‡^<10^−8,^^‡^1.5 × 10^−7^. Source data are provided as a Source Data file. Individual data and mean ± SEM shown in each summary panel.

To test if intrinsic PKG activation regulates CHIP and PQC in vivo, mice expressing cardiomyocyte-restricted tetracycline-inducible phosphodiesterase 5 (mPDE5A/*oe*) were subjected to permanent coronary artery ligation to induce myocardial infarction (MI). In the absence of doxycycline, myocyte PDE5A activity increases 4-fold over controls in SHAM operated mice, and even somewhat more after MI (Fig. 4a, upper). However, in SHAM where PKG activity is low, this has no impact, but after MI when PKG activity normally increases, this is fully inhibited in mPDE5A/*oe* hearts (Fig. 4a, lower). The latter was associated with greater cardiac dilation and worse function as shown by example echocardiograms (Fig. 4b) and ejection fraction (Fig. 4c, upper). Pathological fibrosis (Fig. 4c, lower) and heart and lung weights (Fig. 4d) were also significantly greater in mPDE5A/*oe* hearts after MI.

**Fig. 5:**
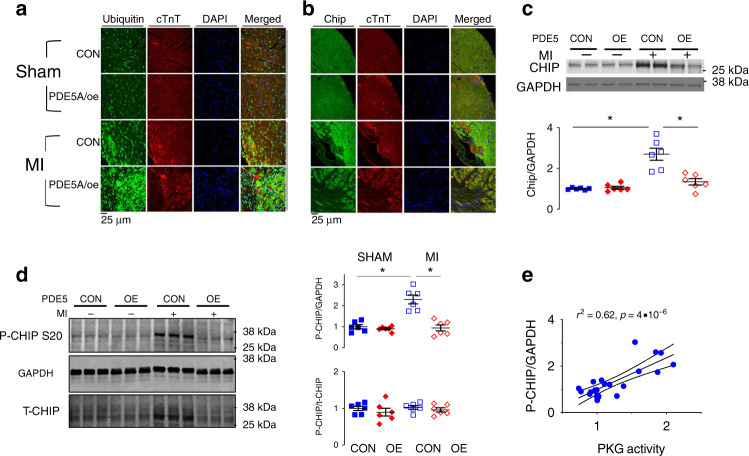
Impact of reduced in vivo myocyte PKG activity on CHIP. **a** Fluorescent confocal images of transverse myocardial sections with antibody staining for ubiquitin (green), cardiac troponin T (cTnT, red), and nuclei (DAPI, blue). There is increased myocyte ubiquitin staining after MI in mPDE5/*oe* myocardium. **b** Fluorescent confocal images of same myocardium stained for CHIP (green), cTnT, and DAPI. Myocyte CHIP expression is reciprocally reduced in mPDE5/*oe* heart after MI. Each examination performed with biological duplicates. **c** Representative Western blot and summary data for total CHIP expression in both experimental groups at baseline (CON) and after MI. *n* = 6 biological replicates, 2WANOVA, *p* = 0.0011 for interaction of group and +/− MI; Tukey mct: *p < 5 × 10^−6^. **d** Example immunoblot and summary results for phospho-S20 CHIP, and ratio of pCHIP to total CHIP in peri-infarct zone before and after MI. *n* = 6 biological replicates; 2WANOVA (*p* = 0.0002 for interaction term); Tukey mct: *p = 10^−5^. **e** Myocardial PKG activity directly correlates with levels of p/t CHIP. Data are from MI study with rest and post MI data pooled in the two groups. Source data are provided as a Source Data file. Individual data and mean ± SEM shown in each summary panel.

Global protein ubiquitination increased in the peri-infarct region after MI, and this was exacerbated in mPDE5A/*oe* mice (Fig. 4e). Confocal fluorescent microscopy showed that following MI, ubiquitinated proteins increased primarily in the interstitium of WT hearts. However, in mPDE5A/*oe* hearts, this accumulated mostly in myocytes (identified by Troponin T co-localization, Fig. 5a), consistent with the myocyte-selective decline in PKG activity in mPDE5A/*oe* hearts. This was paralleled by a decline in CHIP expression in myocytes as revealed by confocal microscopy (Fig. 5b). Total LV myocardial CHIP protein expression increased after MI in controls, but this was prevented in mPDE5A/*oe* hearts (Fig. 5c). Once again, we found CHIP mRNA expression was unaltered (Supplementary Fig. 5) so increased protein levels appeared due to post-translational modifications. We also examined proteasome activity finding it significantly reduced after MI, and further reduced in both SHAM and MI hearts with PDE5A/*oe* to block PKG activity (Supplementary Fig. 6).

Suppressing PKG activation in mPDE5A/*oe* hearts also blunted an increase in S20-CHIP phosphorylation found in the littermate controls (WT) hearts after MI (Fig. 5d). The decrease in both pS20 and total CHIP occurred in parallel, leaving the pS20/total CHIP ratio unaltered. This differs from the dissociation of pS20 from total CHIP upon acute PKG modulation (Fig. 2f), suggesting the two become linked with more chronic PKG suppression. We also found pS20-CHIP levels directly correlated with PKG activity measured in the same myocardium (Fig. 5e). Independent support for depressed pS20 CHIP in mPDE5A/*oe* post-MI myocardium was obtained using mass spectroscopy (Supplementary Fig. 7).

Female mice were also subjected to the same MI stress but displayed much less left ventricular dysfunction and chamber dilation as observed in males. Interestingly, enhanced female-dependent protective effects against MI were associated with both greater CHIP gene and protein levels (Supplementary Fig. 8).

### CHIP S20 phosphorylation augments/stabilizes CHIP-HSC70 binding and post-translational protein stability

**Fig. 6:**
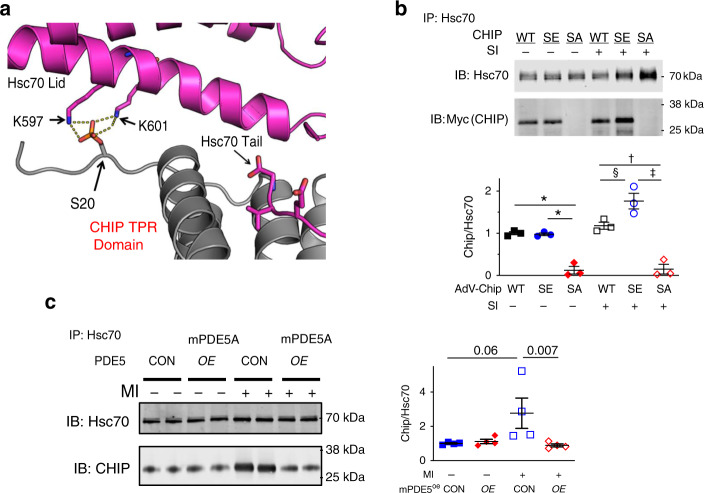
PKG regulates CHIP-chaperone interaction and post-translational stability. **a** Structural model of binding region between Hsc70 lid motif and the TPR region in CHIP. The location of S20 in reference to opposing lysine residues K597 and K601 in Hsc70 is shown. **b** Upper: example immunoprecipitation using Hsc70 as bait, and detecting Myc-tagged WT, SE, or SA CHIP in both control and SI conditions. There is minimal Hsc70-CHIP co-precipitation when CHIP-SA is expressed. Lower: summary data, *n* = 3 biological replicates/group, 1WANOVA for each condition, Holms-Sidaks mct: *p = 1.2 × 10^−4^, ^†^0.003, ^‡^0.0005, ^§^0.02. **c** Immunoprecipitation gels and summary data for Hsc70 and CHIP interaction in vivo with and without MI in mice with mPDE5*oe* and littermate controls (CON). Hsc70 is bait, and antibody detection using CHIP and Hsc70. *N* = 4 biological replicates/group, Kruskal-Wallis test, Dunns mct. *P* values in figure. Source data are provided as a Source Data file. Individual data and mean ± SEM shown in each summary panel.

While CHIP has chaperone-independent activity, its binding to Hsc(Hsp70) is considered central to its functionality for promoting protein degradation^7,8^. S20 is located within the TPR domain of CHIP and coordinates with Hsc70 Lid-domain and the lysine residues (K597, K601) involved in protein-protein interactions (Fig. 6a). The thermodynamic impact of S20 phosphorylation on CHIP-Hsp70 binding was tested by isothermal titration calorimetry of CHIP-WT and CHIP-S20E. CHIP-S20E increased Hsp70 affinity by ~0.5 µM (59%), coupled with a 5.6 kJ mol^−1^ more negative enthalpy (Δ*H;* −26.4 ± 3.3 to −32.1 ± 0.9, *p* < 0.05*)* and a smaller change in entropy (Δ*S*, 25.5 ± 8.3 to 9.8 ± 3.7 J/Kmol, *p* < 0.05) compared to CHIP-WT. The Δ*H* and Δ*S* are consistent with our model based on the published structure of the CHIP-TPR/Hsp70-lid-tail complex^26^ predicting the S20E mutation (or S20 phosphorylation) would enhance the electrostatic interaction between CHIP and the Hsp70 lid domain, with an additional hydrogen bond potentially restricting motion of the lid with respect to CHIP-TPR, decreasing entropy for the CHIP/Hsp70 complex.

To test whether thermodynamic stabilization of CHIP-Hsc70 due to S20 phosphorylation enhances protein binding, lysate from myocytes with or without exposure to SI were immunoprecipitated using Hsc70 as bait. We confirmed co-precipitation of CHIP and Hsc70 in cells expressing CHIP-WT or CHIP-S20E, but this was absent if CHIP-S20A was expressed (Fig. 6b). Reduced CHIP-Hsc70 interaction was also observed in vivo following MI in hearts with PKG activation suppressed by mPDE5A/*oe*, whereas this was enhanced in WT (Fig. 6c).

**Fig. 7:**
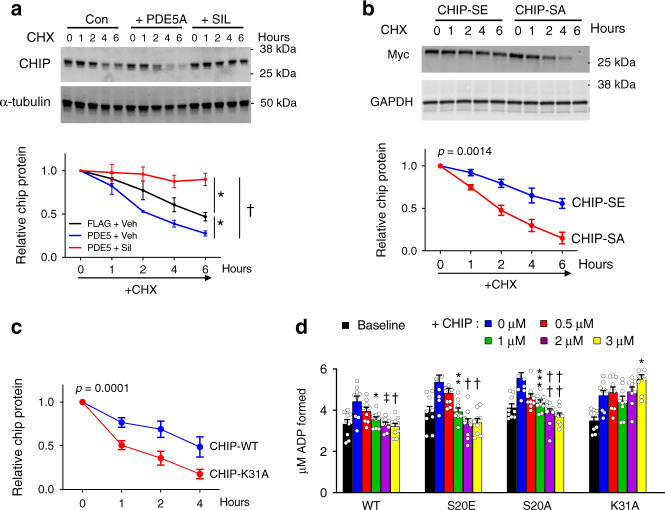
Influence of S20 modification on CHIP post-translational half-life. **a** NRCMs infected with AdV-Flag or AdV-Flag-PDE5 to reduce PKG activation, and with SIL added to enhance PKG activation placed in media containing cycloheximide (CHX, 100 µM) to block protein synthesis and H_2_O_2_ (50 µM) to induce oxidative stress. *N* = 4–5 separate experiments with 5 independent time points per experiment. ANCOVA results for differences between paired relations: *p = 0.001; ^†^0.000009. **b** Same general protocol but with NRCMs over-expressing either S20A or S20E CHIP mutants. *P*-value for slope difference by ANCOVA. **c** Same protocol but WT and K31A CHIP mutants. *P*-value for offset difference by ANCOVA. **d** Hsp70/Hsp40 dependent protein refolding ATPase assay showing capacity of CHIP to reduce activity in a dose dependent manner in wild type (WT), S20A, and S20E CHIP mutants. K31A CHIP expression prevents this negative modulation. *N* = 9 biological replicants, RMANOVA performed within each experiment, Dunnets mct: *p = 0.004; **0.006, ***0.002; ^†^0.0005, ^††^0.0002, ^‡^*p* = 0.002 – each versus no CHIP added (blue bar). Source data are provided as a Source Data file. Regressions (**a**–**c**) and summary plot (**d**) each display mean ± SEM.

The preceding results suggested that phosphorylation of CHIP at S20 enhances its protein half-life. To test this, myocytes were treated with cycloheximide (100 µM) to block protein synthesis and then exposed to hydrogen peroxide (50 µM) as a stress stimulus. Under these conditions CHIP had a protein half-life of ~6 h (Fig. 7a), concordant with prior data^27^. This shortened to ~2 h if PKG activity was blocked by PDE5*oe*, but prolonged if PKG was stimulated by SIL. Using a similar protocol with Myc-tagged CHIP constructs, we found CHIP-S20A protein half-life was significantly shorter than CHIP-S20E (Fig. 7b).

Mutation of CHIP Lys 31 (K30 in humans) to Ala is known to abolish CHIP-chaperone interactions, and with it, the inhibitory effect of CHIP on chaperone ATPase activity required for their protein refolding function^8^. As the impact of this mutation on CHIP half-life was not reported, we examined it here, and find the K30A mutation indeed shortened half-life significantly to levels observed with S20A mutants (Fig. 7c). There was, however, a striking difference between the two mutations with respect to their inhibition of Hsp70 ATPase activity. As expected, Hsp70 activity was dose-dependently inhibited by CHIP-WT, and effect that was lost with CHIP-K31A. However, both CHIP-S20E and S20A displayed similar efficacy as CHIP-WT, the latter despite its disruption of Hsc-70 interaction (Fig. 7d). This indicates that the S20A mutant destabilizes the CHIP and Hsc (Hsp) 70 interaction but does not prevent it, and chaperone activity is still modulated. However, loss of S20 phosphorylation favors CHIP protein degradation, and the reverse increased protein half-life and functionality.

### Knock-in mice expressing CHIP-S20E are protected against MI-proteotoxicity

**Fig. 8:**
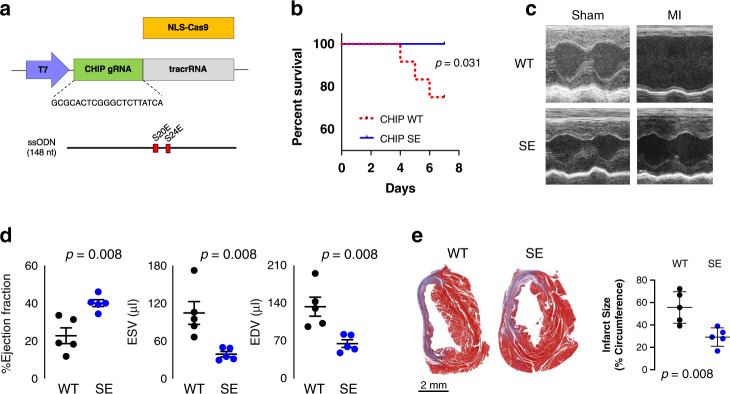
Mice with global S20E KI are protected against myocardial infarction. **a** Guide RNA to target CHIP S20/S24 region, and the replacement RNA that includes a silent mutation of the PAM sequence to further enhance homology directed repair**. b** CHIP^S20E^ (*n* = 10 heterozygous; *n* = 7 homozygous) mice have reduced mortality following myocardial infarction due to total coronary occlusion. **c** Example M-mode echocardiograms show improved wall motion and less heart dilation in CHIP^S20E^ (SE) KI mice versus littermate (WT) controls. **d** Summary data for LV function and volumes from same experiment. *N* = 5 mice for each group, mean ± SEM, *p*-value shown for Mann–Whitney test unpaired test. **e** Whole heart cross-section histology shows larger infarct territory in WT versus CHIP^S20E^ hearts. Summary quantitation (right). *N* = 5 biological replicates, mean ± SD, *p* value for Mann–Whitney unpaired test. Source data are provided as a Source Data file.

**Fig. 9:**
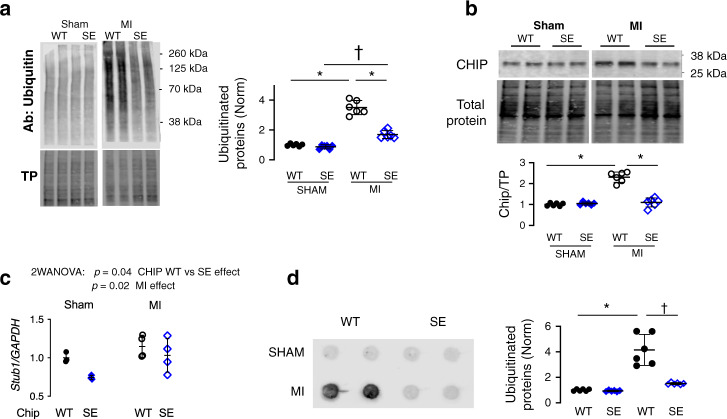
Protein ubiquitination is reduced post MI in CHIP^S20E^ mice. **a** Example protein ubiquitination gel and total protein (TP) loading gel from CHIP^S20E^ and littermate controls (WT) after Sham or MI intervention. Summary results: *n* = 6 biological replicates, 2WANOVA; interaction term *p* = 4 × 10^−7^, Tukey mct: *p ≤ 3 × 10^−9^, ^†^3 × 10^−4^. **b** Example immunoblot for total CHIP protein expression at baseline and post-MI in the peri-infarct zone from WT and CHIP^S20E^ mice. Summary: 6 biological replicates, 2WANOVA, Tukey mct: *p ≤ 5 × 10^−10^. **c** CHIP gene expression (*Stub1/Gapdh*) same study measured in Sham and post MI mice. 2WANOVA results provided in figure. **d** Filter trap assay for insoluble proteins probed for ubiquitin which are increased after MI in WT but maintained at low levels in CHIP^S20E^ myocardium. *N* = 6 biological replicates, 2WANOVA, Tukey mct: *p = 12 × 10^−7^, ^†^*p* = 1.9 × 10^−6^. Source data are provided as a Source Data file. Individual data and mean ± SD shown in each summary panel.

To test whether S20 phosphorylation of CHIP confers cardioprotection by enhancing PQC in vivo, we generated global CHIP^S20E^ KI mice using CRISPR/Cas9 (Fig. 8a). Mice are born in normal Mendelian ratios and exhibit normal resting cardiac function and lifespan (Supplementary Table 1). We then tested if expression of CHIP^S20E^ protects against cardiac damage/dysfunction following myocardial infarction, focusing on males as they displayed more severe post-MI disease. Compared to littermate controls CHIP^S20E^ (both homozygotes and heterozygotes) subjected to MI exhibited similar lower mortality (Fig. 8b). Echocardiograms showed preserved function in CHIP^S20E^ mice after MI (Fig. 8c, d), and reduced infarct size (Fig. 8e). These improvements were accompanied by reduced accumulation of ubiquitinated proteins (Fig. 9a). Whereas CHIP protein level rose after MI in controls, it was unaltered in KI mice (Fig. 9b), perhaps reflecting 100% of the mutant CHIP being pseudo-phosphorylated. CHIP^S20E^ gene expression was slightly lower than WT (Fig. 9c), that might reflect longer CHIP protein half-life. To test for CHIP-S20E enhanced functionality, we determined if the mutation influenced clearance of insoluble protein aggregates. Insoluble protein aggregates were trapped by dot blot filter assay and then stained for ubiquitin. CHIP-S20E hearts had a marked clearance of ubiquitinated proteins after MI (Fig. 9d). Thus, expression of CHIP-S20E at endogenous levels is cardioprotective after MI in vivo and improves proteostasis.

## Discussion

The capacity of CHIP to enhance the clearance of damaged/misfolded proteins and suppress proteotoxicity is relevant to immunogenicity^28^, aging^29^, metabolic stress^12^, and many human diseases including cardiac and neurological^10,16,30,31^. Here we reveal a mechanism for post-translational augmentation of CHIP protein levels and functionality by means of PKG-mediated phosphorylation at serine 20. The result is a cascade of effects: increased interaction with co-chaperone Hsp (Hsc) 70, increased post-translational protein half-life resulting in a net rise in protein levels, and greater clearance of insoluble ubiquitinated protein aggregates in conditions of proteotoxic stress. This can provide a more rapid response to counter such stress that leverages kinase modification rather than requiring de novo gene and protein synthesis. To our knowledge, the CHIP^S20E^ KI mouse is the first point-mutation in vivo model involving CHIP. The potency of this modification is revealed by the substantial gain-of-function it confers to counter infarction injury even in heterozygotes. Our results have therapeutic implications, given the growing role played by CHIP in cardiac, skeletal muscle, neurogenerative, and oncologic disease, and clinical armamentarium available to stimulate PKG.

CHIP pS19 (human equivalent of S20 in mouse) is found in human phospho-proteomic data bases^20,21^, however, we uncovered only one prior study examining any impact^32^. This study in mice identified CHIP-pS20 in a kinase-interactome screen for cyclin-dependent kinase 5 (CDK5), and reported that it disrupted CHIP binding to truncate apoptosis-inducing factor (AIF) leading to neuronal apoptosis. The prior study did not assess whether pS20 altered CHIP protein levels, chaperone binding, or any broader functionality, nor was there in vivo confirmation. The CHIP S20E KI mouse expresses this mutation in all cells, yet we observe no detrimental neurological or other effects. CDK5 is expressed in the heart but in contrast to PKG, is not activated by myocyte stress^33^. Most importantly, we find consistent protective effects from pS20 CHIP associated with increased protein levels and chaperone interaction.

The current data support a linkage between CHIP S20 phosphorylation and its enhanced binding to Hsc70 and post-translational protein level. Expression of the K31A mutant, which also prevents CHIP-chaperone binding markedly reduced post-translational half-life similar to that seen with the S20A mutant. The measured increased biophysical interaction for CHIP-S20E and associated increased protein stability further supports this. However, unlike K31A mutants that prevent chaperone interaction, the S20A mutation did not alter Hsp70/Hsp40 complex ATPase activity, a hallmark of CHIP-mediated inhibition of refolding activity. Thus, the proteins must still engage. Our cell results with CHIP-S20A indicate that while Hsc70 interactions may not be detectable by under the stringent IP conditions, they are not functionally eliminated.

While this study focused on the impact of PKG on CHIP, studies have also shown the kinase modifies many other pathways relevant to myocardial pathological signaling and remodeling^34^, and these could also have contributed to the net effects observed. Among these mechanisms are PKG-mediated mitochondrial protection and cytoprotection against ischemia/infarction^35^, activating the regulator of G-protein signaling proteins RGS2 and RGS4 to inhibit Gq-receptor coupled signaling, blocking transient receptor potential canonical channel 6 to blunt fibrosis and hypertrophy^36,37^, and activating tuberous sclerosis complex 2 (TSC2) to blunt the mechanistic target of rapamycin complex-1 (mTORC1) stimulating autophagy and reducing pathological growth^25^. Specific to the UPS, PKG activates the 26S proteasome enhancing degradation of damaged/misfolded proteins in models of heart disease and neurodegenerative^22–24^. The current study confirms this, but also shows PKG has a constitutive role since genetic suppression of its activity in both myocytes and intact hearts depressed proteasome activity. While an impact from these alternative PKG effectors cannot be ignored, it is intriguing that preventing S20 phosphorylation of CHIP was itself sufficient to blunt PKG-mediated protection in myocytes. Moreover, the opposite—expressing the S20E form to mimic PKG modulation was very protective by itself in cells and intact hearts against ischemic stress. This indicates that CHIP-PKG interaction is important and influential, particular in settings where PQC is sub-optimal and its enhancement beneficial.

Both CHIP and Hsc70 are involved in chaperone-mediated and chaperone-assisted selective autophagy^38,39^, and whether and how these roles are impacted by S20 modification remains to be determined. CHIP interacts with the proteasome and the lysosome for substrate degradation and is itself ubiquitinated^6–9^, however the regulators of CHIP protein degradation remain uncertain. Further studies are needed to determine the mechanisms regulating the turnover of CHIP, the degradation system responsible (e.g., proteasome, lysosome, or both), and how the lack of S20 phosphorylation (e.g., S20A) accelerates CHIP recycling. There are feedback loops involved in CHIP binding to heat shock element of heat shock factor-1, a transcriptional protein that, once activated by CHIP, increases transcription of Hsps such as Hsp70^40^. Whether pS20 modifies this also remains to be tested. Here we determined the S20 site regulates binding between CHIP and its primary cognate chaperone Hsc70 which is structurally near identical to Hsp70. Additional studies are needed to test if the interaction between CHIP and other co-chaperones (e.g., Hsp90, Bag1, Bag3) are also regulated by S20 phosphorylation. Oxidative stress reduces CHIP protein half-life^41^ and also depresses the functionality of myocardial PKG1α^42^. Whether these events are also linked remains to be tested. Lastly, the full scope of kinases that can modify CHIP S20 remains to be determined, though PKG is likely to be relevant in many tissues.

The intersection of PKG signaling with enhanced CHIP functionality is important, since from a clinical perspective, it suggests new therapeutic targets suitable for an array of existing and developing PKG activation strategies. Clinically used agents include nitric oxide donors, organic nitrates, soluble guanylate cyclase stimulators, and PDE5 inhibitors that enhance NO-dependent cGMP synthesis, and natriuretic peptides (NP), neprilysin inhibitors, and PDE9 inhibitors that increase NP-dependent cGMP stimulation^43–45^. All of these targets have existing small molecules or peptides in clinical use and/or under active studies in humans with cardiac or neurological disease. While PKG activation has historically been leveraged for its vasodilator capacity, the present data reveal its role in PQC by enhancing CHIP functionality. Both PKG and CHIP are widely expressed in many cell types, including the brain and cancer cells, and thus we believe the current findings will likely apply to the heart as well as other tissues, providing novel therapeutic opportunities.

## Methods

### Mouse models

Studies used mice (C57BL6/J background) with myocyte-specific PDE5A over-expression under control of an α-MHC tetracycline-inducible promoter^46^ and their littermate controls. We also generated a new global knock-mouse expressing CHIP S20E/S24E (SE) using CRISPR/Cas9 targeting/insertion methods (Transgenic Mouse Core Laboratory, Johns Hopkins University). CHIP guide RNA was designed using an algorithm developed by Ranganathan^47^, and subsequently cloned into [a T7 expression vector]. In vitro transcription for the guide RNA was achieved with the NEB HiScribe T7 High Yield RNA Synthesis kit, and subsequent purification with the Ambion MEGAclear Transcription Clean-up Kit. Cas9 protein was from PNA Bio, and ssODN for the S20E/S24E point mutations from Integrated DNA Technologies. C57Bl/6 blastocyst injections were performed with a mix consisting of: 30 ng/μl Cas9 protein, 12.5 ng/μl guide RNA, and 20 ng/μl ssODN. Two founders were generated, and were subsequently backcrossed into C57Bl/6J mice to eliminate potential off-target mutations from Cas9 editing. The colony was maintained by breeding heterozygous males and heterozygous females to generate knock-in homozygous, knock-in heterozygous, and wild-type littermates at a 25:50:25 ratio. Custom PCR genotyping primers are:

SE Forward: GGCGGCGAGCCTGATAAGGAG

WT Forward: GGCGGCAGCCCTGATAAGAGC

Reverse: CCTAGACTCGGGAACAGCAATCCGG

### Myocardial infarction (MI)

Male and female mice (aged ~3–4 months) were subjected to transmural myocardial infarction by mid-left anterior descending artery ligation under open thoracotomy^48^. Controls underwent open chest surgery and LAD isolation without ligation. Data obtained 1-week after infarction included 2D echocardiography (Vevo2100, 20-MHz; Visualsonics) in conscious mice to measure LV volume and ejection fraction, with analysis blinded to study group. The protocol was approved by the Johns Hopkins Animal Care and Use Committee.

### Cultured cardiomyocyte studies

Primary cultures of NRCMs were prepared as described^45^. After overnight culture, subsets of NRCMs were infected with adenovirus (AdV) containing a FLAG-tagged PDE5, FLAG control, Myc-tagged CHIP WT or K31A (provided by Dr. Cam Patterson), S20E or S20A (Welgen Inc., Worcester, MA) (10MOI). Forty-eight hours after infection, cells were exposed to either simulated ischemia (SI) or normal conditions. SI involved incubating cells in DMEM containing deoxyglucose (20 mM), NaCl (125 mM), KCl (8 mM), KH_2_PO_4_ (1.2 mM), MgSO_4_ (1.25 mM), CaCl_2_ (1.2 mM), NaHCO_3_ (6.25 mM), sodium lactate (5 mM), and HEPES (20 mM) with a pH of 6.6. Cells were then placed in an incubator chamber (Billups-Rothenberg) with 95% nitrogen, 4% carbon dioxide, and 1% oxygen. Normoxic controls were incubated in DMEM supplemented with 10% FBS in air composed of 95% carbon dioxide and 5% oxygen. RNA or protein was harvested after 48 hours of SI. Cells were also treated with sildenafil (1 µM) or vehicle, or small interference RNA (siRNA, 100 pM) specific for rat *Stub1* (Qiagen, Cat. #: SI01532251) or scrambled control provided 24 h after plating and transfected with Xfect (Takara) per manufacturer protocol.

### Protein analysis

Tissue and cultured cardiomyocytes were lysed in 1X lysis buffer (Cell Signaling Technology), protein concentration was measured by bicinchoninic acid assay (Pierce), and extracts were subjected to SDS-PAGE using Novex Tris-Glycine Gels (4–20% gradient gel, Life Technologies) or Mini-protean TGX Gels (4–20% gradient gel, Bio-Rad), transferred to nitrocellulose membranes, and probed with various primary antibodies from Cell Signaling Technology: CHIP (#2080S), Hsc70 (#8444S), Myc (#2276S), GAPDH (#2118S), and α-tubulin (#3878S), AbMart Inc.: p-CHIP S20 (Custom antibody, Shanghai, China; Project: 25011–1), Sigma: ubiquitin antibody (#SAB4503053), and Li-Cor: fluorescence-labeled secondary antibodies (#926-32211, 926-32210, 926-68023, or 926-68022). Gels were imaged (Odyssey, Li-Cor) and band intensity quantified (Odyssey Software 3.1).

### Proteasome activity assay

Snap-frozen tissues and cultured NRCMs were homogenized on ice in cytosolic extraction buffer (50 mM Tris-HCl pH 7.5, 250 mM Sucrose, 5 mM MgCl_2_, 0.5 mM EDTA, and 1 mM DTT). Protein concentrations were determined with bicinchoninic acid (BCA) reagents (Pierce) and equally concentrated in proteasome assay buffer (50 mM Tris-HCl pH 7.5, 40 mM KCl, 5 mM MgCl_2_, and 1 mM DTT). Chymotrypsin-like, trypsin-like, and caspase-like activities were determined in the presence of 28 μM (chymotrypsin) or 14 μM (trypsin and caspase) ATP, utilizing the following fluorogenic substrates: Suc-LLVY-AMC (18 μM, Boston Biochem #S280), Ac-RLR-AMC (45 μM, Boston Biochem #S290), and Z-Leu-Leu-Glu-AMC (40 μM, Boston Biochem #S230), respectively. The plate was read at an excitation wavelength of 380 nm and an emission wavelength of 460 nm using a Spectramax M5 (Molecular Devices). Activity was combined, using a 50:25:25 ratio for relative contribution of chymotrypsin, trypsin, and caspase-like activities^49^.

### LDH/MTT cytotoxicity assay

Lactate dehydrogenase (LDH) leakage and (3-[4,5-dimethylthiazol-2-yl]-2,5-diphenyltetrazolium bromide) (MTT) were assessed in cultured cardiomyocytes and media after SI from the same culture dish. LDH was assayed by commercial assay (Roche) following manufacturer’s protocol, read in 96-well microplates on a Spectramax M5 (Molecular Devices) at 490 nm absorbance. For MTT, cells were washed in phosphate-buffered saline, MTT (Sigma-Aldrich) working solution added to the culture dish, incubated at 37 °C, solubilized with acidic isopropanol, transferred to a 96 well plate, and read at 570 nm with background subtraction at 650 nm on a Spectramax M5.

### Cycloheximide (CHX) chase assay

NRCMs were incubated in serum-free DMEM containing 10 μM hydrogen peroxide to induce oxidative stress and 100 μM CHX (Sigma-Aldrich) to block further protein synthesis, as described^22^. Cells were harvested at consecutive time points (e.g., 0, 1, 2, 4, and 6 h) after CHX and whole-cell lysates analyzed by immunoblot for CHIP or Myc *(Myc*-CHIP).

### Tissue histology/immunostaining

The myocardium was fixed in 4% paraformaldehyde, paraffin embedded and sectioned into 4 μm slices, and stained with Masson’s Trichrome. Fibrosis was quantified by color-based pixel count and intensity in parallel sections of the ischemic and remote areas (Aperio Image Scope). Transmural infarct size was estimated as the arc angle subtended by dense scar in a mid-MI cross section divided by 360° and expressed as percent. Immunostaining for ubiquitin (Sigma) and CHIP (Cell Signaling) was performed using AlexaFluor 488 (#A-11008 Lot#1345061, used at 1:500) conjugated secondary antibodies following the manufacturer’s protocol. Counterstain was performed using Troponin-T (#A-11004 used at 1:500) (Thermo Scientific) antibodies and DAPI (Invitrogen #S36968). Image acquisition was performed on a Leica confocal microscope (TCS SPE II) at ×40 magnification.

### Quantitative real-time PCR

Total RNA was extracted with Trizol Reagent (Invitrogen) from myocardium or isolated myocytes per manufacturer’s instructions, then reverse transcribed into cDNA using a High Capacity RNA-to-cDNA Kit (Applied Biosystems, Life Technologies). cDNA was subjected to PCR amplification using either TaqMan specific primers (Applied Biosystems) for CHIP (*Stub1*, mouse #Mm00490634_m1, rat #Rn01751757_g1) or gylceraldehyde-3-phosphate dehydrogenase (*GAPDH*, mouse #99999915_g1, rat #Rn01775763_g1) (Applied Biosystems). The Biorad CFX384 qPCR machine was used to determine the threshold cycle (Ct) values determined by crossing point method, were normalized to the respective housekeeping gene GAPDH (Applied Biosystems) values for each run.

### PKG and PDE5A activity assay

In vitro PKG activity was measured by colorimetric assay (Cyclex), and PDE5A activity by fluorescence polarization assay (Molecular Probes) following manufacture protocols of cell lysates from either from heart tissues or NRCMs. PKG activity is assessed by detecting phosphorylation of a pseudo-substrate, while PDE5 activity is measured by fluorescence polarization assay under linear conditions with or without PDE5A inhibitor (sildenafil 1 μM).

### Recombinant proteins for in vitro assays

Human HSP40 (DnaJB1), human CHIP and CHIP point mutants in pET30a with a C-terminal His-tag and hHsc70 (HSPA8) wild type with an N-terminal His-tag in pCOLA-Duet1 were expressed in BL21(DE3) and purified on Ni-NTA (Qiagen). Hsc70 and CHIP proteins were induced and expressed for 22 h at 18 °C. Hsp40 protein was induced and expressed for 5 h at 25 °C. Required stock solutions of proteins were prepared by diluting high concentrated stocks in protein storage buffer into protein storage buffer, 20 mM HEPES (pH 7.4), 150 mM NaCl. Human, active PKG1α purified from *Sf*9 cells was purchased (SRP0371, Sigma).

### PKG and CHIP/CHIP-S20A in vitro kinase assays

Aliquots of recombinant CHIP or PKG1α were diluted to either 12.5 μM or 0.25 μM in Kinase Dilution Buffer II (K23-09, SignalChem: 5 mM MOPS, pH 7.2, 2.5 mM β-glycerol-phosphate, 5 mM MgCl2, 1 mM EGTA, 0.4 mM EDTA, 50 ng/μl BSA, 0.5 mM DTT). A 250 µM stock solution (5X) of ATP was made in Kinase Assay Buffer I (K01-09 SignalChem: 25 mM MOPS, pH 7.2, 12.5 mM β-glycerol-phosphate, 25 mM MgCl2, 5 mM EGTA, 2 mM EDTA, 0.25 mM DTT). A 100 μM cGMP solution was purchased (#G47-09, SignalChem). The final buffer composition of complete reactions was 10.5 mM MOPS, 4 mM Beta-glycerol-phosphate, 8 mM MgCl2, 1.6 mM EGTA, 0.64 mM EDTA, 0.08 mM DTT, 10 µM cGMP, 50 µM ATP. CHIP or PKG1α were used at final concentrations of 2.5 μM or 0.1 μM, respectively (25:1 molar ratio). Reactions were started with the addition of the 5X ATP solution, and incubated for 30 °C for 1 h. Aliquots were either flash frozen for phospho-proteomic analysis or used for reducing SDS-PAGE/immunoblot analysis. Controls included no substrate (CHIP) or no kinase (PKG1α). Experimental triplicates were run on multiple days to confirm findings.

Mass spectrometry was performed at the UNC-CH Proteomics Center (Chapel Hill, NC, USA) as described^50^. Samples were reduced, alkylated and digested with trypsin overnight. After digestion, peptides were desalted using C18 spin columns and dried down via vacuum centrifugation. Each sample was analyzed in technical duplicate by LC/MS/MS using the Easy nLC 1200-QExactive HF (Thermo). Data were searched against a Uniprot *E.Coli* database (Uniprot *E.Coli* proteome ID (UP000000625) available at), appended with human CHIP-WT, CHIP-S19A, and PKG1α sequences, using Sequest within Proteome Discoverer (v2.1). Data were filtered using an FDR of 1% and peak areas were mean normalized.

### Isothermal titration calorimetry

Isothermal titration calorimetry was carried out using a TA Instruments NanoITC equilibrated at 25 °C and utilizing a stir speed of 600 rpm. Experiments were conducted in triplicate and data represent the average ± one standard deviation. Titrations utilized 350 µM WT or S20E CHIP titrated into 50 µM Hsp70 (385–641) as a series of twenty-four 2 µL injections*.*

### Chaperone refolding ATPase activity assay

Human HSP40 (DnaJB1), human CHIP and CHIP point mutants in pET30a with a C-terminal His-tag and hHsc70 (HSPA8) wild type with an N-terminal His-tag in pCOLA-Duet1 were expressed in BL21(DE3) and purified on Ni-NTA (Qiagen). Hsc70 and CHIP proteins were induced and expressed for 22 h at 18 °C. Hsp40 protein was induced and expressed for 5 h at 25 °C. Required stock solutions of proteins were prepared by diluting high concentrated stocks in protein storage buffer into protein storage buffer, 20 mM HEPES (pH7.4), 150 mM NaCl. Hsc70 and Hsp40 were used at a concentration of 0.5 µM and 0.25 µM, respectively, with a gradient of CHIP protein concentrations (0.5–6 µM). Hsp40-stimulated ATPase activity of Hsc70 was measured using fluorescent polarization with the Transcreener® ADP2 FP Assay (BellBrook Labs, 3010-1 K) on a Clariostar plate reader (BMG) in 384 well black, non-binding plates (Greiner, 784900). Optimized instrument settings were used: Excitation wavelength: 590 nm; Emission wavelength: 675 nm; Required value: 5%; Target mP: 20; Gain A: 1824; Gain B: 1854; Focal Height: 12.4 mm. Values of µM ADP formed in the reactions were interpolated from an ATP/ADP standard curve run in parallel.

### Statistical analysis

Data are presented by the individual experimental results along with mean ± SEM. Data were processed with Microsoft Excel Version 16. Group comparisons were performed by 1- or 2-way ANOVA. Multiple comparisons testing was performed by Holm-Sidak or Tukey’s test using Prism 8 software. Data that were not normally distributed were assessed by non-parametric analysis (Kruskal Wallis) test and Dunn’s test for multiple comparisons. Sample sizes and individual statistical results for all analyses are provided in the figures or legends.

### Reporting summary

Further information on research design is available in the Nature Research Reporting Summary linked to this article.

## Supplementary information

Supplementary Information

Reporting Summary

## Data Availability

The authors declare that the data supporting the findings of this study are available within the paper and its supplementary information files. Source data are provided with this paper. Any remaining data that support the results of the study will be available from the corresponding author upon reasonable request. The mass spectrometry proteomics data have been deposited to the ProteomeXchange Consortium via the PRIDE partner repository with the dataset identifier PXD021531.

## Source data

Source Data
