# Supplementary material for: CHIP phosphorylation by protein kinase G enhances protein quality control and attenuates cardiac ischemic injury
Source: Nat Commun. 2020 Oct 20;11:5237. doi: 10.1038/s41467-020-18980-x (PMC7575552; doi:10.1038/s41467-020-18980-x)
Supplement: Supplementary file 1 — Supplementary Information [file 41467_2020_18980_MOESM1_ESM.pdf]

Supplementary Figures and Table

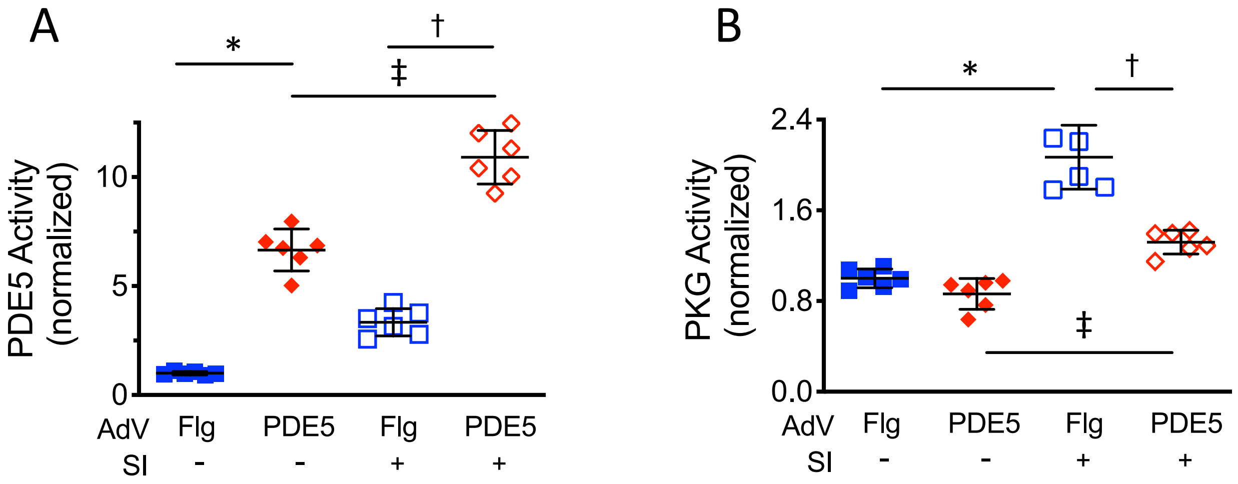

Supplementary Figure 1.

PDE5A overexpression blocks PKG activation with simulated ischemia (SI).

A) Myocytes are infected with adenovirus expressing Flag or Flag-PDE5A and then subjected to normal conditions or 48 hours of simulated ischemia. PDE5A activity is increased by overexpression in both conditions but is higher after SI. N=6 biological replicates/group, 2WANOVA, Tukey multiple comparisons test (mct): * $p=1.3{\cdot}10^{-9}$, † $7{\cdot}10^{-12}$ ‡ $1.6{\cdot}10^{-7}$. **B)** This is accompanied by suppression of PKG activation particularly after SI. N=6 biological replicates/group; 2WANOVA, Tukey mct: * $p=4.4{\cdot}10^{-9}$, † $1.3{\cdot}10^{-6}$ ‡ $8.4{\cdot}10^{-4}$. Data are mean ± SD for both panels.

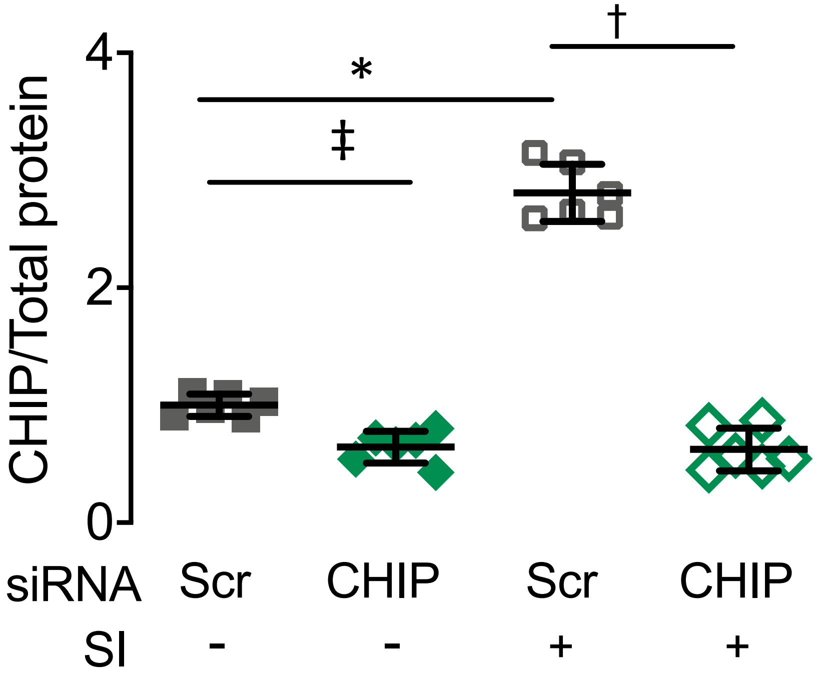

Supplementary Figure 2.

Summary data for efficacy of CHIP siRNA in isolated cardiomyocytes.

Quantitation of immunoblots (Figure 1E) showing efficacy of siRNA to reduce CHIP protein levels in cardiomyocytes in vitro. N=6 biological replicates, 2WANOVA, Tukey mct: * $p=10^{-12}$, † $<10^{-12}$ ‡ 0.01. Mean ± SD.

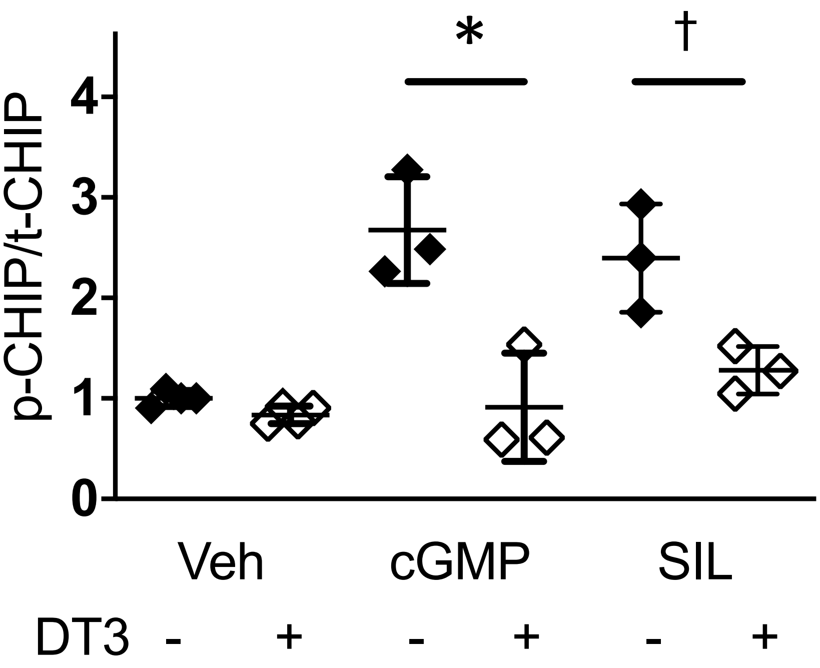

Supplementary Figure 3.

PKG activation is required for cGMP and PDE5 inhibition to phosphorylate CHIP.

Quantitation of immunoblots as displayed in Figure 2F. Data are ratio of phospho (pS20)/total (p-CHIP/t-CHIP) normalized to values for Vehicle control without PKG inhibitor DT3. The ratio increases with either cGMP stimulation or inhibiting of PDE5, and this is reversed by co-incubation with PKG inhibitor DT3. 3-4 biological replicates per condition. 2WANOVA, P=.00025 for DT3 effect; Sidak's mct: * p=0.029; † 0.001. Mean ± SD.

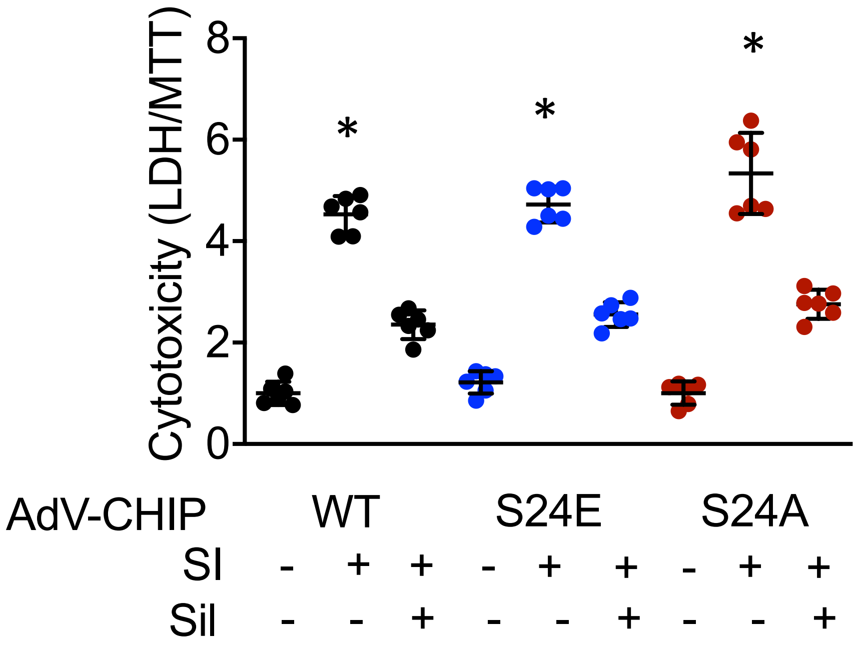

Supplementary Figure 4.

Minimal effect from mutating CHIP S24 on myocyte response to simulated ischemia (SI).

Mutating CHIP S24 to a phosphomimetic (S24E) or null (S24A) has minimal impact on cytotoxic response to SI or benefit from PKG activation using PDE5 inhibitor sildenafil (SIL). N=6 biological replicates/group; 1WANOVA within each CHIP genotype group, Sidak's mct: * $p<10^{-11}$ versus other two conditions in each respective group. Mean ± SD.

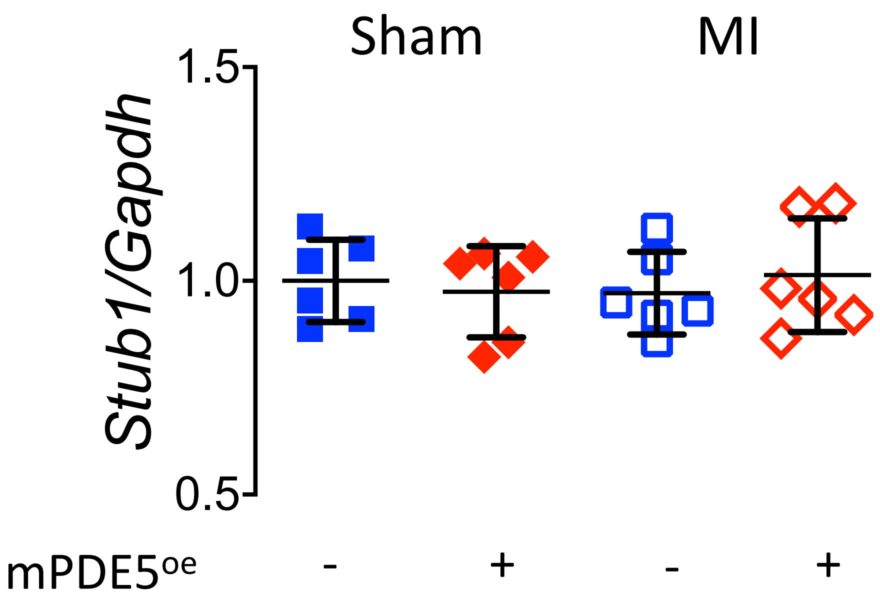

Supplementary Figure 5.

CHIP gene expression (*Stub1)* normalized to GAPDH is unaltered in both control and mPDE5oe mice with and without myocardial infarction.

Myocardial mRNA expression for CHIP (*Stub1/Gapdh*) in mice with myocyte specific PDE5A overexpression (mPDE5^{oe}) or littermate controls with sham surgery or myocardial infarction (MI). There is no change in gene expression in these conditions. N=6 mice for each group, 1WANOVA, p=0.89. Mean ± SD.

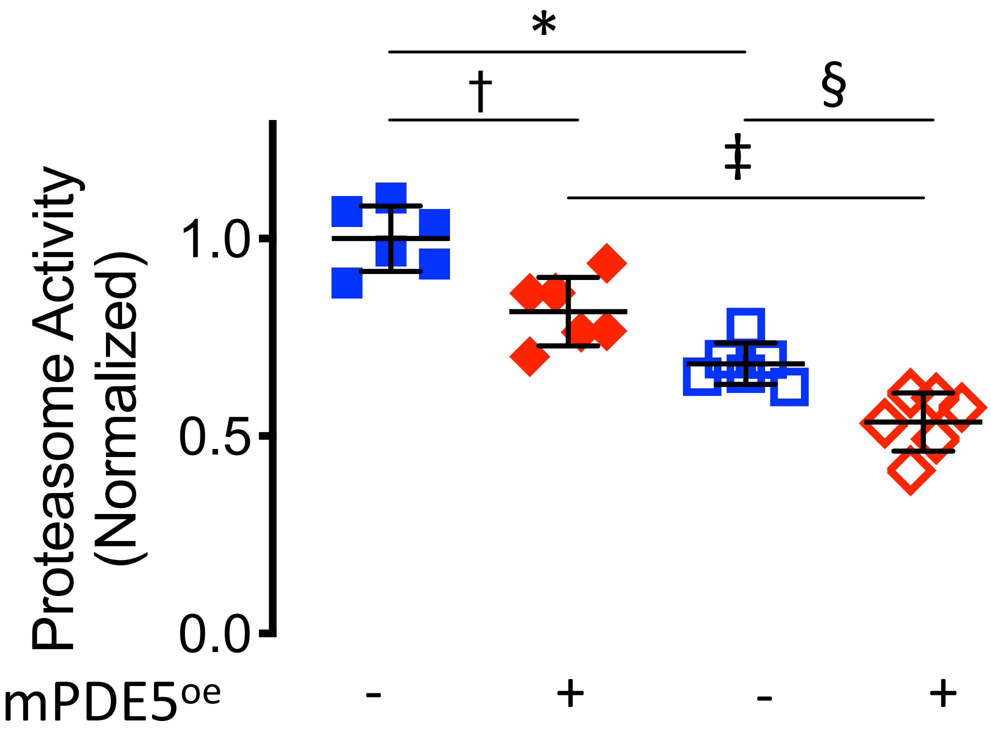

Supplementary Figure 6.

Proteasome activity in myocardium of sham or MI mice +/- mPDE5^{oe}.

Proteasome activity in peri-infarct region from WT and mPDE5A*oe* hearts in normal and post-MI conditions. PDE5A overexpression reduced proteasome activity similarly in mice with or without the infarct. N=6 biological replicates; 2WANOVA, no interaction (p=0.55); * $2 \cdot 10^{-6}$, † 0.001; ‡ 10^{-5}, § 0.006. Mean ± SD.

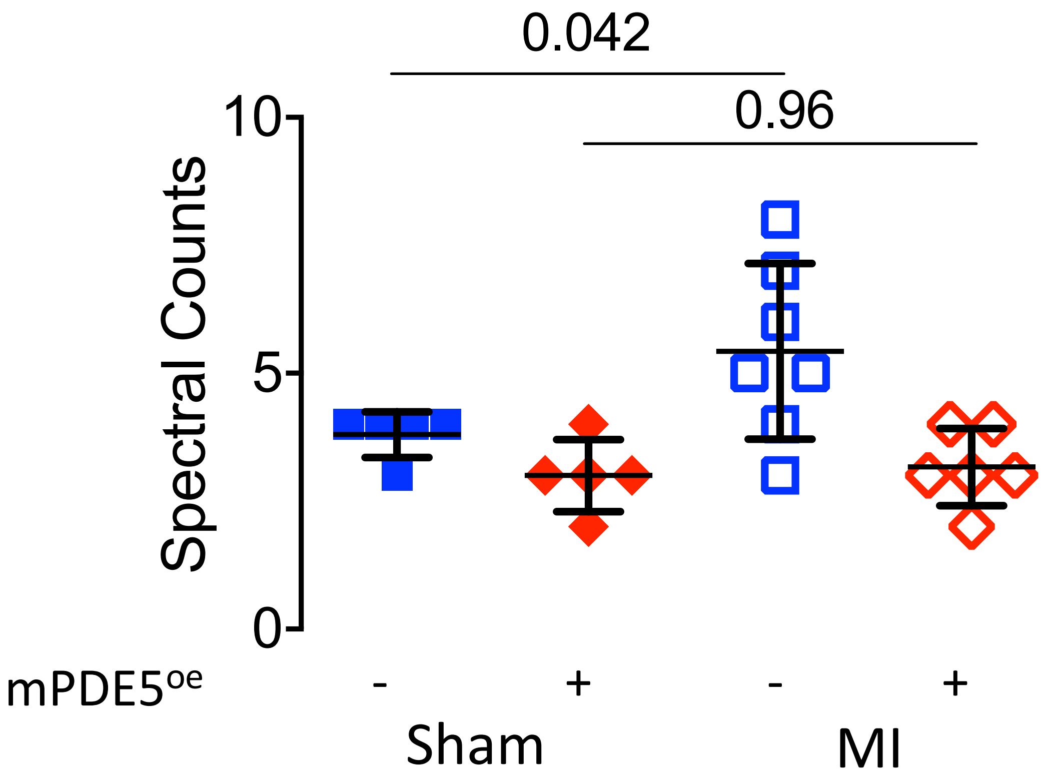

Supplementary Figure 7.

Mass spectroscopic detection of phospho-serine 20 on CHIP protein.

Myocardial extracts for sham and MI from the anterior wall (peri-infarct for MI group) in mPDE5A*oe* and littermate control mice. n=5-6 different animals/group; 2WANOVA, effect of mPDE5^{oe} =0.005; Sidak's mct: p values in figure. Mean ± SD.

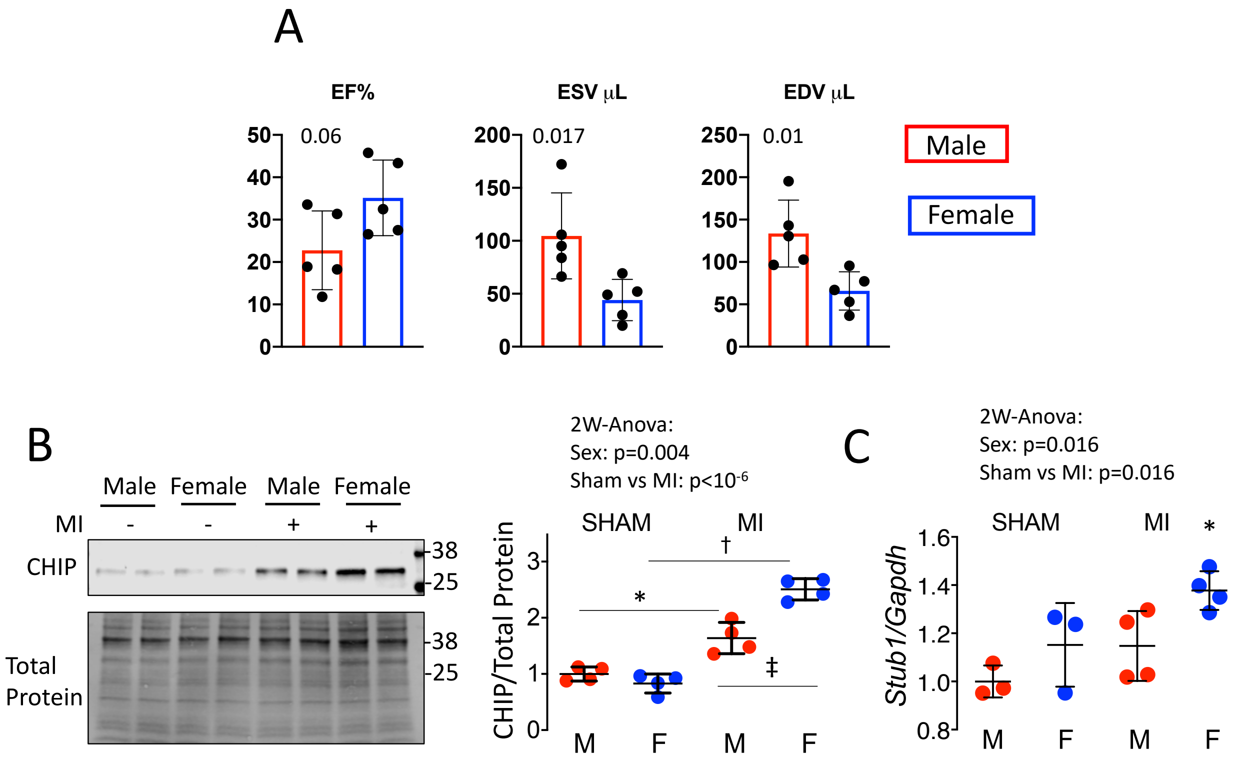

Supplementary Figure 8. Sex disparities in cardiac functional parameters and in CHIP (gene and protein) levels following MI.

A) Female mice subjected to MI display significantly less myocardial dysfunction and chamber dilation as compared to males. N=5 different mice per group. P-values shown in each plot are for unpaired t-test between groups. **B)** Example immunoblot for CHIP expression (left) and summary data (right) determined in female versus male myocardium with or without MI. Protein levels were greater in females than males after MI. N=4 biological replicates per group. 2WANOVA (interaction of sex and condition: $2 \cdot 10^{-4}$, Tukey mct: * p=0.003; † $2.6 \cdot 10$-7, ‡ $2.3 \cdot 10^{-4}$. **C)** CHIP gene expression (*Stub1/Gapdh)* in males versus females. N=3-4 mice in each group. 2WANOVA (sex effect p=0.016), Sidak's mct: * p=0.048. All panels data are Mean ± SD.

Supplementary Table 1. Resting cardiac function and left ventricular mass from 9-12 months old $CHIP^{S20E/S20E}$ mice and littermate WT controls (n=5-7/group). The KI mice show normal heart function and morphometry (p values for 2-tailed T-test). Data are mean ± SD.

	Mice Aged 9-12 Months				
	**Chip WT**	**n**	**Chip SE**	**n**	**p-value**
**Body Weight (g)**	29.8 ± 2.3	6	27.7 ± 2.5	7	0.14
**Heart Weight (mg)**	164.3 ± 14.9	6	147.0 ± 19.1	7	0.10
**Left Ventricular Weight (mg)**	119.9 ± 9.1	6	108.1 ± 13.6	7	0.10
**Lung Weight (mg)**	145.9 ± 17.1	6	131.8 ± 12.9	7	0.12
**Heart/Body Weight (mg/g)**	5.5 ± 0.3	6	5.3 ± 0.3	7	0.15
**Left Ventricular/Body Weight (mg/g)**	4.0 ± 0.2	6	3.9 ± 0.2	7	0.27
**Lung/Body Weight (mg/g)**	4.9 ± 0.4	6	4.8 ± 0.2	7	0.47
**Heart Rate (bpm)**	701.7 ± 24.7	5	733.1 ± 59.6	5	0.31
**Ejection Fraction (%)**	66.9 ± 3.6	5	67.0 ± 2.1	5	0.95
