# Supplementary material for: CHIP phosphorylation by protein kinase G enhances protein quality control and attenuates cardiac ischemic injury
Source: Nat Commun. 2020 Oct 20;11:5237. doi: 10.1038/s41467-020-18980-x (PMC7575552; doi:10.1038/s41467-020-18980-x)
Supplement: Supplementary file 2 — Reporting Summary [file 41467_2020_18980_MOESM2_ESM.pdf]

nature research

Corresponding author(s): David A. Kass, MD

Last updated by author(s): Aug 30, 2020

Reporting Summary

Nature Research wishes to improve the reproducibility of the work that we publish. This form provides structure for consistency and transparency in reporting. For further information on Nature Research policies, see our Editorial Policies and the Editorial Policy Checklist.

Statistics

For all statistical analyses, confirm that the following items are present in the figure legend, table legend, main text, or Methods section.

n/a	Confirmed	
☐	☒	The exact sample size (*n*) for each experimental group/condition, given as a discrete number and unit of measurement
☐	☒	A statement on whether measurements were taken from distinct samples or whether the same sample was measured repeatedly
☐	☒	The statistical test(s) used AND whether they are one- or two-sided<br>*Only common tests should be described solely by name; describe more complex techniques in the Methods section.*
☐	☒	A description of all covariates tested
☐	☒	A description of any assumptions or corrections, such as tests of normality and adjustment for multiple comparisons
☐	☒	A full description of the statistical parameters including central tendency (e.g. means) or other basic estimates (e.g. regression coefficient) AND variation (e.g. standard deviation) or associated estimates of uncertainty (e.g. confidence intervals)
☐	☒	For null hypothesis testing, the test statistic (e.g. *F*, *t*, *r*) with confidence intervals, effect sizes, degrees of freedom and *P* value noted<br>*Give P values as exact values whenever suitable.*
☒	☐	For Bayesian analysis, information on the choice of priors and Markov chain Monte Carlo settings
☒	☐	For hierarchical and complex designs, identification of the appropriate level for tests and full reporting of outcomes
☒	☐	Estimates of effect sizes (e.g. Cohen's *d*, Pearson's *r*), indicating how they were calculated

Our web collection on statistics for biologists contains articles on many of the points above.

Software and code

Policy information about availability of computer code

Data collection	All data were collected on individual hardware devices (e.g. Visualsonics Vevo 2100 for echocardiographic images, or Licor Odyssey for Western blot and Odyssey Software Version 3.1, Biorad CFX3 84 qPCR machine for mRNA expression, Seaquest with proteome discoverer Version 2.1 was used for phospho-proteomic analyses. No stand-alone open source or commercial software was used for data collection.
Data analysis	Mouse genetic background assessment utilized SNaP-MaP''' and Map-Synth''' performed at the Geisel School of Medicine at Dartmouth University, Hannover, NH; Western blot images were performed using Licor Image Studio Software 3.1, echocardiographic images were performed using VisualSonics Vevo 2100, data was processed using Microsoft Excel Version 16, and statistical analysis using Graphpad Prism Version 8.

For manuscripts utilizing custom algorithms or software that are central to the research but not yet described in published literature, software must be made available to editors and reviewers. We strongly encourage code deposition in a community repository (e.g. GitHub). See the Nature Research guidelines for submitting code & software for further information.

Data

Policy information about availability of data

All manuscripts must include a data availability statement. This statement should provide the following information, where applicable:

- Accession codes, unique identifiers, or web links for publicly available datasets
- A list of figures that have associated raw data
- A description of any restrictions on data availability

The authors declare that all data supporting the findings of this study are available within the paper and its supplement data source file. Mouse and cell models are available from the corresponding author upon request. Uniprot E.Coli proteome ID (UP000000625) available at https://www.uniprot.org/proteomes/UP000000625.

Field-specific reporting

Please select the one below that is the best fit for your research. If you are not sure, read the appropriate sections before making your selection.

- [x] Life sciences
- [ ] Behavioural & social sciences
- [ ] Ecological, evolutionary & environmental sciences

For a reference copy of the document with all sections, see nature.com/documents/nr-reporting-summary-flat.pdf

Life sciences study design

All studies must disclose on these points even when the disclosure is negative.

Sample size	Based on our prior experience with relevant publications were cited in the manuscript such as Ranek et al. Nature 2019 and Rainer et al. Circ Res. 2014. The various assays both in vitro and in vivo, there are generally at least 6 experimental/biological replicates for every assay and condition. Power analyses were conducted on the results of pilot experiments to determine the sample size required to obtain statistically significant results. As revealed throughout the study, the results were generally very robust with group data often not overlapping, and statistical differences highly significant with this sample size.
Data exclusions	No data were excluded.
Replication	All assays were successfully replicated multiple times by independent investigators - see sample size - with at least 6 replicates.
Randomization	There was no treatment protocol that required a placebo control-randomization procedure. The two in vivo studies involved genetic models in which either PDE5A or CHIP were modified, with wild type mice serving as the control. These mice were randomly assigned to sham or myocardial infarction surgery.<br>Randomization for the cell studies occurred by plating all cells at the same time in equal density and under the same conditions. Plates were then randomly divided between experimental and control groups.
Blinding	The surgeon performing the myocardial infarction or sham surgery was blinded as to genetic model; the echosonography who imaged the hearts and performed the M-mode analysis was also blinded as to the genetic model or animal condition. Investigators were blinded during tissue collection and processing and were not unblinded until is was time to run the molecular assays. Molecular assays based on tissue from these mice were performed by individuals who were not blinded as to the tissue source in order to allow for proper controls to be present in every experiment (e.g. WT sham mice). These assays were conducted by individual investigators. Similar procedure was in place for cell studies.

Reporting for specific materials, systems and methods

We require information from authors about some types of materials, experimental systems and methods used in many studies. Here, indicate whether each material, system or method listed is relevant to your study. If you are not sure if a list item applies to your research, read the appropriate section before selecting a response.

Materials & experimental systems

n/a	Involved in the study	
☐	☒	Antibodies
☐	☒	Eukaryotic cell lines
☒	☐	Palaeontology and archaeology
☐	☒	Animals and other organisms
☒	☐	Human research participants
☒	☐	Clinical data
☒	☐	Dual use research of concern

Methods

n/a	Involved in the study	
☒	☐	ChIP-seq
☒	☐	Flow cytometry
☒	☐	MRI-based neuroimaging

Antibodies

Antibodies used	The following primary antibodies were used in this study from- Cell Signaling Technology: GAPDH #2118 clone 14C10lot 10 used at 1:1,000, CHIP #2080 clone C3B6 lot 5 used at 1:1,000, Myc-tag #2276 clone 9B11 used at 1:1,000, HSPA8 #8444 clone D12F2, and α-tubulin #3873 clone DM1A lot 12 used at 1:1,000; Abmart- phospho-CHIP (S20) used at 1:500; Sigma: ubiquitin #SAB4503053 lot 310385 used at 1:1,000; Thermofischer- Alexafluor 488 goat anti-rabbit (#A-11008 Lot#1345061, used at 1:500) and Alexafluor 568 goat anti-mouse (#A-11004 used at 1:500), and a Li-Cor: total protein stain #926-11016 lot C80522-02 used at 5 ml/membrane.<br><br>All antibodies used were either well established in the literature, for which validation in the model system was previously determined.
Validation	All antibodies purchased from commonly used companies (Cell Signaling Technology, Sigma, AbMart, and Li-Cor), which performed the validation. All validation statements, citations, antibody/antigen details are found on the companies websites.

The antibody for S20 Chip phosphosphorylation obtained from Abmart was previously reported (Kim et al. Cell Death and Differentiation. 2016). Validation was performed in cells expressing of the protein with and without site mutations at the S20 that impact phosphorylation of the residue (e.g. S20A which prevents it) (Figure 2E), and then with and without kinase activation (PK) (Figure 2F).

Cell Signaling Technology validation statement: Western blotting remains one of the most common scientific methods for monitoring protein expression in cells or tissue. The accuracy of western blot results relies heavily of the quality of the primary antibody employed in the immunoblotting. Cell Signaling Technology (CST) provides the highest quality primary and secondary antibodies available for western blotting. CST™ antibodies are produced in-house and validated extensively according to a rigorous protocol.

Sigma validation statement: The success of any immunodetection experiment depends on the quality of the antibodies which are employed. However, antibody reagents vary significantly and when selecting an antibody for a downstream application it is a good idea to spend some time ensuring that not only has it been tested in the chosen experimental setup but that it also demonstrates the required specificity, sensitivity and reproducibility. This valuable data is generated during the antibody manufacturing and validation process and can be found on the product datasheet with which the antibody is supplied.

AbMart validation statement: AbTarget™ delivers high quality, validated monoclonal antibodies against a large number of proteins in a cell, organ, tissue, organelle or any complex protein sample. AbTarget™ is a fit-for-purpose, reverse antibody methodology by generating proteome-scale antibody libraries first, isolation and target identification second. This approach has been proven to be highly effective in yielding applications (WB/IF/flow cytometry/IP) validated antibodies at a proteome-scale. AbTarget™ is based on MabArray™ antibody array technology, a massively parallel, proprietary antibody array platform with close to 50,000 pre-made monoclonal antibodies. MabArray™ represents a fundamental breakthrough by solving the long-standing content problem of antibody array technology.

Li-Cor validation statement: In quantitative Western blotting (QWB), normalization mathematically corrects for unavoidable sample-to-sample and lane-to-lane variation by comparing the target protein to an internal loading control. The internal loading control is used as an indicator of sample protein loading, to correct for loading variation and confirm that observed changes represent actual differences between samples. Revert 700 Total Protein Stain is used to assess sample protein loading in each lane as an internal loading control. After transfer and prior to immunodetection, the membrane is treated with this near-infrared fluorescent protein stain and imaged. Membrane staining can verify that sample protein was uniformly loaded across the gel, and assess the quality and consistency of protein transfer.

Thermofisher validation statement: Antibodies are some of the most critical research reagents used in the lab. Poor specificity or application performance can significantly frustrate the ability to obtain good results, which can cause critical delays. Underperforming antibodies result in a lack of reproducibility, wasting time and money. In other words, researchers need antibodies that bind to the right target and work in their applications every time. To help ensure superior antibody results, we've expanded our specificity testing methodology using a 2-part approach for advanced verification. This helps ensure the antibody will bind to the correct target. Our antibodies are being tested using at least 1 of the following methods to ensure proper functionality in researcher's experiments. Click on each testing method below for detailed testing strategies, workflow examples and data figure legends.

Knockout—expression testing using CRISPR-Cas9 cell models
Knockdown—expression testing using RNAi to knockdown gene of interest
Independent antibody verification (IAV)—measurement of target expression is performed using two differentially raised antibodies recognizing the same protein target
Cell treatment—detecting downstream events following cell treatment
Relative expression—using naturally occurring variable expression to confirm specificity
Neutralization—functional blocking of protein activity by antibody binding
Peptide array—using arrays to test reactivity against known protein modifications
SNAP-ChIP™—using SNAP ChIP to test reactivity against known protein modifications
Immunoprecipitation-Mass Spectrometry (IP-MS)—testing using immunoprecipitation followed by mass spectrometry to identify antibody targets
Part 2—Functional application validation

These tests help ensure the antibody works in a particular application(s) of interest, which may include
(but are not limited to):

Western blotting
Flow cytometry
ChIP
Immunofluorescence imaging
Immunohistochemistry
Most antibodies were developed with specific applications in mind. Testing that an antibody generates acceptable results in a specific application is the second part of confirming antibody performance.

Advanced Verification

The Advanced Verification badge is applied to products that have passed application and specificity testing. Advanced Verification testing of Invitrogen antibodies continues to expand across the portfolio. An antibody lacking the badge should be seen as an antibody that has not yet undergone testing – not a reflection of the specificity of the product. This badge can be found in the search results and at the top of the product specific webpages. Data supporting the Advanced Verification badges can be found in product specific data galleries.

Eukaryotic cell lines

Policy information about cell lines

Cell line source(s)	We used mouse embryonic fibroblasts (standard cell line, or MEFs with CHIP knocked-out) provided from the laboratory of Jonathan Schisler. These cells were originally isolated in the laboratory of Dr. Cam Patterson (mentor and collaborator of Dr. Jonathan Schisler) as described (Min et al. Molecular and Cellular Biology. 2008. DOI: 10.1128/MCB.00296-08), have been used in many prior studies, and are well established models. Other cells are primary isolated neonatal ventricular myocytes from rat - so not a cell line per se. These cells are isolated from rats that were purchased from Charles River. Methods for this preparation are well established and referenced.
Authentication	We did not perform specific authentication procedures for the cells used in this study, as the cell lines are well established and widely used, and the primary isolations are not cell lines.
Mycoplasma contamination	Our cultured cells are assessed for evidence of bacterial contamination in a more general manner, as part of maintenance of our incubator systems and being sure we have not infectious contamination. However, we did not specifically test for mycoplasma.
Commonly misidentified lines (See ICLAC register)	None were used.

Animals and other organisms

Policy information about studies involving animals; ARRIVE guidelines recommended for reporting animal research

Laboratory animals	For all in vivo studies the mice were aged around 2-3 months at the time of pressure overload or sham surgeries. Wild type C57 black mice (male and female) were purchased from Charles River. CHIP SA and SE knock in mice (male and female were designed by our lab and generated at the Johns Hopkins Mouse Transgenics Core using CRISPR/Cas9 technology. They are also raised in the C57 black background. Mice were housed in the Johns Hopkins Mouse Facility with 12 hour light/dark cycles, 65-75 degrees Fahrenheit, 40-60% humidity, and on site veterinarian care.
Wild animals	The study did not involve the use of wild animals
Field-collected samples	The study did not involve the use of samples collected from the field.
Ethics oversight	All protocols and procedures were approved by the Johns Hopkins IUCAC. The studies were in compliance with all ethical regulations. Both male and female mice were utilized in this study.

Note that full information on the approval of the study protocol must also be provided in the manuscript.
